# Germline pathogenic variants in *HNRNPU* are associated with alterations in blood methylome

**DOI:** 10.1038/s41431-023-01422-9

**Published:** 2023-07-05

**Authors:** Sunwoo Lee, Eguzkine Ochoa, Magdalena Badura-Stronka, Deirdre Donnelly, Damien Lederer, Sally A. Lynch, Alice Gardham, Jenny Morton, Helen Stewart, France Docquier, Fay Rodger, Ezequiel Martin, Ana Toribio, Eamonn R. Maher, Meena Balasubramanian

**Affiliations:** 1 Department of Medical Genetics, University of Cambridge, Cambridge, CB2 0QQ UK; 2 Poznan University of Medical Sciences, Poznań, Poland; 3 Northern Ireland Regional Genetics Centre, Belfast Health and Social Care Trust/City Hospital, Belfast, Northern Ireland UK; 4 Centre for Human Genetics, IPG, Charleroi, Belgium; 5 Department of Clinical Genetics, Our Lady’s Children’s Hospital, Crumlin, Dublin Republic of Ireland; 6 London North West University Healthcare NHS Trust Genetics Service, Middlesex, UK; 7 West Midlands Regional Clinical Genetics Service and Birmingham Health Partners, Birmingham Women’s and Children’s Hospitals NHS Foundation Trust, Birmingham, UK; 8 Oxford Centre for Genomic Medicine, Oxford University Hospitals NHS Foundation Trust, Oxford, UK; 9 Stratified Medicine Core Laboratory NGS Hub, Cambridge Biomedical Campus, Cambridge, UK; 10 Department of Oncology & Metabolism, University of Sheffield, Sheffield, UK; 11 Sheffield Clinical Genetics Service, Sheffield Children’s NHS Foundation Trust, Sheffield, UK

**Keywords:** DNA methylation, Metabolic disorders, Genetics research

## Abstract

*HNRNPU* encodes a multifunctional RNA-binding protein that plays critical roles in regulating pre-mRNA splicing, mRNA stability, and translation. Aberrant expression and dysregulation of *HNRNPU* have been implicated in various human diseases, including cancers and neurological disorders. We applied a next generation sequencing based assay (EPIC-NGS) to investigate genome-wide methylation profiling for >2 M CpGs for 7 individuals with a neurodevelopmental disorder associated with *HNRNPU* germline pathogenic loss-of-function variants. Compared to healthy individuals, 227 *HNRNPU*-associated differentially methylated positions were detected. Both hyper- and hypomethylation alterations were identified but the former predominated. The identification of a methylation episignature for *HNRNPU*-associated neurodevelopmental disorder (NDD) implicates *HNPRNPU*-related chromatin alterations in the aetiopathogenesis of this disorder and suggests that episignature profiling should have clinical utility as a predictor for the pathogenicity of *HNRNPU* variants of uncertain significance. The detection of a methylation episignaure for *HNRNPU*-associated NDD is consistent with a recent report of a methylation episignature for *HNRNPK*-associated NDD.

## Introduction

Advances in genomics have resulted in increasingly large numbers of genes being identified as causing neurodevelopmental disorders (NDDs) [1, 2]. *HNRNPU* encodes a component of a multiprotein complex that binds heterogeneous nuclear RNA and scaffold-attached DNA [3, 4]. Other members (*n* = 32) of the large heterogeneous nuclear ribonucleoprotein family that have been implicated in human disease include *HNRNPH1,* *HNRNPH2,* *HNRNPK, HNRNPR* and *SYNCRIP* [5]. Following suggests that inactivation of *HNRPNU* might contribute to the neurological and neurodevelopmental phenotype of 1q34q44 microdeletion syndrome [6, 7] as de novo mutations in *HNRNPU* were reported in rare cases of epileptic encephalopathy [8, 9]. Subsequently, the phenotype associated with pathogenic variants in *HNRNPU* was extended to include early-onset seizures, severe intellectual disability, speech impairment, hypotonia, microcephaly and ventriculomegaly [10–13]. Dysmorphic features (high arched eyebrows, long palpebral fissures, overhanging columella, widely spaced teeth and thin upper lip) have also been described [11, 12].

Interpreting the potential pathogenicity of variants of uncertain significance (VUSs) remains a major challenge in many areas clinical genetics, including the diagnosis of NDDs [14–16]. A major cause of NDDs are variants in chromatin modifying genes (CMGs) (e.g., histone lysine methyltransferases or histone acetylases etc.) and for many of these disorders, evidence of disordered epigenetic regulation can be detected through alterations of DNA methylation patterns (episignatures) in peripheral blood [2, 17]. The identification of CMG-associated NDD specific episignatures can be used to aid variant interpretation and suggest candidate CMGs in unsolved NDDs [15, 17–22]. In addition to a role in posttranscriptional RNA processing, *HNRNPU* (also known as scaffold attachment factor A (SAF-A)) is also reported to have roles in gene transcription, maintenance of higher-order chromatin structure and X-inactivation via Xist [23–25]. Recently, a methylation episignature was described for Au-Kline syndrome, a NDD associated with germline mutations in *HNRNPK* [16]. In the light of this finding, we investigated whether *HNRNPU*-related NDD was associated with a methylation episignature.

## Subjects and methods

We performed genome-wide methylation profiling of >2 M CpGs with a targeted next generation sequencing assay (Illumina TruSeq^®^ Methyl Capture EPIC NGS) as described previously [17]. Written informed consent was obtained for all participants and the study was approved by South Birmingham Research Ethics Committee.

Genomic DNA with *HNRNPU* pathogenic mutations (*n* = 7) were extracted from whole blood by standard methods. Bisulfite conversion, library preparation, target enrichment and sequencing (Illumina NextSeq 2000) were performed at the Cambridge University Department of Medical Genetics Stratified Medicine Core Laboratory (SMCL) as described previously [17]. Raw methylation beta-values were extracted by RnBeads R package (https://rnbeads.org). Data pre-processing and bioinformatics analysis, and detection and visualisation of methylation episignatures were performed according to our standard procedure (see Lee et al. [17, 26]). If a significant batch effect (age, gender, batch-based) was detected, the target variables were adjusted by surrogate variable analysis (SVA) using the sva package. The *p*-value of differentially methylated sites was determined either by a two-sided Welch test or by a linear model employed in the *limma* package, and the combined *p*-values (for CpG islands) were determined by Fisher’s method. During the process, neighbouring CpGs combined together and assigned as ‘DMB (differentially methylated blocks).’ DMBs were combined based on their functional similarity. Only DMBs (including CpG Islands) with a *p*-value lower than 0.01 and a methylation difference between controls and diseases group of more than 20% were considered significant for genome-wide CpG site methylation analysis. A summary of the sequencing coverage and sequencing reads is included in Supplementary Table 1.

## Results

### Clinical and genetic features of *HNRNPU* patient cohort

**Fig. 1:**
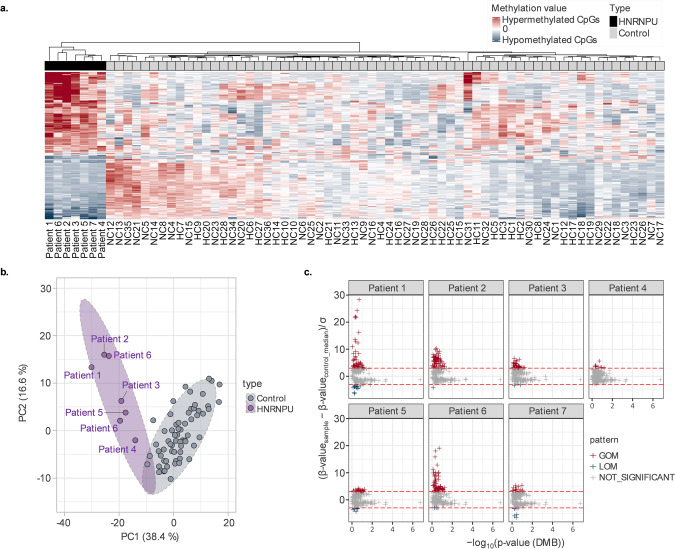
*HNRNPU*-specific methylation episignature. **a** The genome-wide methylation episignatures of *HNRNPU* samples were determined by calculating the mean normalised methylation beta-value relative to the control group. Unsupervised clustering analysis revealed a clear separation between the 7 patients and the control group, with approximately 55% of differentially methylated positions (DMPs) exhibiting hypermethylated profiles and approximately 44% showing hypomethylation. **b** To eliminate potential biases introduced by normalised data, a PCA clustering analysis was performed based on preprocessed beta-values. The results demonstrated that 227 DMPs were able to effectively differentiate the *HNRNPU* group from the control group. **c** Scatter plots were generated by comparing the methylation beta-values of individuals with the mean values of the healthy control group, using a confidence interval of ±3 standard deviations (3 SD). A significant pattern of the number of DMPs with gain of methylation (GOM) compared to loss of methylation (LOM) DMPs was detected (17.6% hypermethylated, 2.08% hypomethylated).

The seven individuals studied had been diagnosed with a *HNRNPU*-NDD after the identification of a germline *HNRNPU* variant (see Table 1). All *HNRNPU* variants were assessed as likely pathogenic or pathogenic and were predicted to have a loss-of-function effect (6 were predicted, in the absence of nonsense-mediated mRNA decay, to result in a truncated gene product and one patient was predicted to have *PBRM1* haploinsufficiency as a result of a de novo deletion of exons 1–11) (Table 1). The positions of the truncating variants are plotted on the *HNRNPU* protein in Fig. 1.

**Table 1:** Genetic characteristics of patients with *HNRNPU* variants.

Patient ID	Age at DNA sampling	Sex	Variant Details (*HNRNPU* NM_004501.3)	Protein effect	Reported previously
Patient 1	2	Male	de novo deletion of exons 1–11	Haploinsufficiency	Patient-14 (Taylor et al., 2022 [27])
Patient 2	23	Female	de novo c.847_857del, p.(Phe283Serfs*5)	Truncating	Patient-13 (Durkin et al., 2021 [12])
Patient 3	12	Female	de novo c.23_24delTAinsA, p.(Val8Glufs*4)	Truncating	Patient-4 (Yates et al., 2017 [11])
Patient 4	24	Male	de novo c.1450 C > T, p.(Arg484*)	Truncating	Patient-3 (Durkin et al., 2021 [12])
Patient 5	3	Male	de novo c.1624dup p.(Gln542Pro*8)	Truncating	Not published
Patient 6	22	Female	de novo c.706_707del, p.(Glu236Thrfs*6)	Truncating	Patient-20 (Durkin et al., 2021 [12])
Patient 7	2	Male	de novo c.2365 C > T, p.(Arg789*)	Truncating	Not published

The frequency of the clinical features displayed by the 7 individuals with a *HNRNPU* variant is summarised in Table 1. The overall frequency of clinical features such as seizures, developmental delay, intellectual disability, and hypotonia (Table 2) in the study cohort was similar to that in a previously reported series [27] of 17 patients with *HNRNPU*-NDD (1 patient from the current cohort were also represented in this previous series). Table 3 provides detailed overview of clinical characteristics of current (*n* = 7) cohort with an update on those patients previously published in Taylor et al. and Durkin et al. [12, 27].

**Table 2:** Frequencies of clinical features in *HNRNPU*-associated neurodevelopmental disorder.

Clinical Features	Frequency (%) in current series (*n* = 7)	Frequency (%) in Taylor et al. (*n* = 17)
Seizures	100	100
Global developmental delay	100	100
Intellectual disability	100	94
Dysmorphism	100	94
Hypotonia	100	88
Neonatal hypotonia	28	65
Neonatal feeding difficulties	14	59
Autistic features	57	50
Cardiac abnormality	14	44
Abnormality on brain MRI	28	38

**Table 3:** Clinical characteristics of patients in current cohort with *HNRNPU* variants.

	Patient 1	Patient 2	Patient 3	Patient 4	Patient 5	Patient 6	Patient 7
Decipher ID (in DDD cohort)		DDD-270453	DDD-268390	DDD-305034		DDD-279875	
Sex	Male	Female	Female	Male	Male	Female	Male
Age of DNA sampling (decimal age in years)	2	23	7	24	3	22	2
*HNRNPU* Variant							
Heterozygous cDNA change	Deletion exons 1–11	c.847_857del	c.23_24del	c.1450 C > T	c.1624dup	c.706_707del	c.2365 C > T
Amino acid change	Not applicable	p.Phe283SerfsTer5	p.Val8Glufs*4	p.Arg484Ter	p.Gln542ProfsTer8	p.Glu236Thrfs*6	p.Arg789*
Inheritance	De novo	De novo	De novo	De novo	De novo	De novo	De novo
Additional genetic defect	No	No	No	No	No	No	No
Pregnancy/delivery							
Gestational age at birth (weeks)	40 + 2	37	38	40	39	41 + 2	40
Birth weight (kilograms)	3.7	2.85	2.41	2.63	2.76	2.68	3.7
Neonatal concerns							
Hypotonia	No	Yes	No	No	No	Yes	No
Feeding difficulties	No	Yes	No	No	No	No	No
Other	No	No	No	No	Hypoglycaemia, jaundice, concerns with thermoregulation	No	No
Development							
Intellectual disability	Yes	Yes	Yes	Yes	Yes	Yes	Too young
Mild/Moderate/Severe	Moderate	Severe	Severe	Moderate	Mild	Moderate-severe	
Global dev. delay	Yes	Yes	Yes	Yes	Yes	Yes	Yes
Language delay	Yes	Yes	Yes	Yes	Yes	Yes	Yes
Age of first words	23 months	36 months	5 years	18 months	24 months	24 months	absent
Motor delay	Yes	Yes	Yes	Yes	Yes	Yes	Yes
Age of sitting unsupported	14 months	15 months	2–2.5 years	10 months	9 months	NK	
Age of first steps	3 years	3 years	5 years	20 months	30 months	24 months	22 months
Behavioural Features							
Any psychiatric diagnosis	No	Yes	Very sociable	No	Autism spectrum disorder	Difficult behaviour	Autism spectrum disorder
Other psychopathology	No	Episodic hyperventilation with apnoea, cyanotic episodes in between	hand flapping		No	No formal diagnosis	
Neurological							
Hypotonia	Yes	Yes	Yes	Yes	Yes	Yes	No
Epilepsy	Yes	Yes	No	Yes	Yes	Yes	Yes
EEG abnormality	Yes	Yes	No	Yes	No	Yes	No
MRI-brain	Normal	Abnormal	Normal	Not performed	Abnormal	Normal	Normal
Delayed myelinisation	No	No	No		No		No
Corpus callosum	No	No	No		No		No
Colpocephaly	No	No	No		No		No
Ventriculomegaly	No	No	No		Yes		No
Other	Normal MRI	Minor frontal atrophy	Optic nerve hypoplasia		Periventricular gliosis foci		Normal
Seizures	Yes	Yes	Yes	Yes	Yes	Yes	Yes
Age at first seizure	11 months, febrile	12 months	One seizure only		16 months		24 months
Seizure type	Tonic-clonic	Tonic, tonic-clonic	febrile		Febrile seizures		Atypical febrile, Generalised tonic clonic, absences
Refractory seizures	Yes	Yes	No	No	No		No
Cardiac abnormalities	No	No	none	No	No	Atrial septal defect	No
Renal abnormalities	No	No	none	No	No	No	Vesicoureteral reflux grade 4, surgery
Dysmorphism	Yes	Yes	Yes	Yes	Yes	Yes	Yes
Eyes	No	Strabismus	Epicanthic folds	Elongated palpebral fissures	Normal	Upslanting palpebral fissures	Normal, arched eyebrows
Nose	No	Prominent nasal bridge	Upturned nose	Flat nasal bridge	Depressed nasal base	Flat nasal bridge	Normal
Mouth	Thin upper vermilion		Smooth philtrum, think upper vermilion	Thin upper vermilion	Downturned corners of mouth	Thin upper vermilion	Large
Ear	No	No	Low-set, posteriorly rotated ears	No	Low-set ears with fistula	No	Pretragal tag (right)
Forehead	No	No	No	No	No	No	Frontal bossing
Other	Single palmar crease, sacral dimple	Drooling, short 4/5 metacarpals, rhizomelic shortening	Bilateral 2/3 syndactyly	Hirsutism, broad thumb	Valgus knees and feet	Broad thumbs	
Other clinical features	Hypermetropia, VSDbilateral undescended testes	Hand wringing, bruxism	Drooling, recurrent urinary tract infections, short stature, cyclical vomiting, squint	Type 1 diabetes mellitus, Barrett’s oesophagus, bilateral undescended testes	Stereotypical hand movements and poor eye contact	Scoliosis, pes planus, cold feet	Partial growth hormone deficiency. Short stature. Respond to treatment with growth hormone
Tests performed in past	Epilepsy targeted panel	DDD Trio WES	DDD Trio WES	DDD Trio WES			
Karyotyping	No	Yes	Yes	Yes	Yes	Yes	No
Array	No	Yes	Yes	Yes	Yes	Yes	Yes
Single gene tests	No	Yes	Yes - UBE3A & Angelman testing	Yes	No	Yes	No (had fragile X)
Metabolic testing	No	No	No	No	No	No	Yes
Muscle biopsy	No	No	No	No	No	No	No
Other							SHOX MLPA
Current medical treatment	Levetiracetam	Sodium Valproate, Lamotrigine	Growth hormone injections		Valproate	No treatment at present	Valproate/ Clobazam

### CpG methylation profile

We identified 227 *HNRNPU* specific methylation episignature with an adjusted *p*-value (*p* < 0.01) and a methylation difference between controls (*n* = 64) and *HNRNPU* group (*n* = 7) of more than 20% (Fig. 1). The principal component analysis (PCA) of unsupervised clustering revealed that *HNRNPU* group samples are distinguished from healthy controls based on their methylation episignature (Fig. 1b). More specifically, by analysing the methylation beta-values of patients with the mean values of ±3 standard deviation (3 SD) confidence interval from healthy individuals, a significant gain or loss of methylation was observed (see Fig. 1c) with a slightly higher level of hypermethylation patterns across DMRs (227 DMRs of 7 samples (nDMP=1,589)) which showed 17.6% hypermethylated, 2.08% hypomethylated. Moreover, patient 1 (de novo del in exon 1–11), patient 2 (de novo c.847_857del, p.(Phe283Serfs*5)), and patient 6 (c.706_707del, p.(Glu236Thrfs*6)) exhibited similar hypermethylated patterns in 2 CpG islands (Grch37:chr8:145749856-145750410 and Grch37:chr16:89632593-89632799) while milder hypermethylated signatures observed in other patients; patient 3 (de novo c.23_24delTAinsA, p.(Val8Glufs*4)), patient 4 (de novo c.1450 C > T, p.(Arg484*)), patient 5 (de novo c.1624dup p.(Gln542Pro*8)), and patient 7 (c.2365 C > T p.(Arg789*)) based on hierarchical clustering in Fig. 1a.

**Fig. 2:**
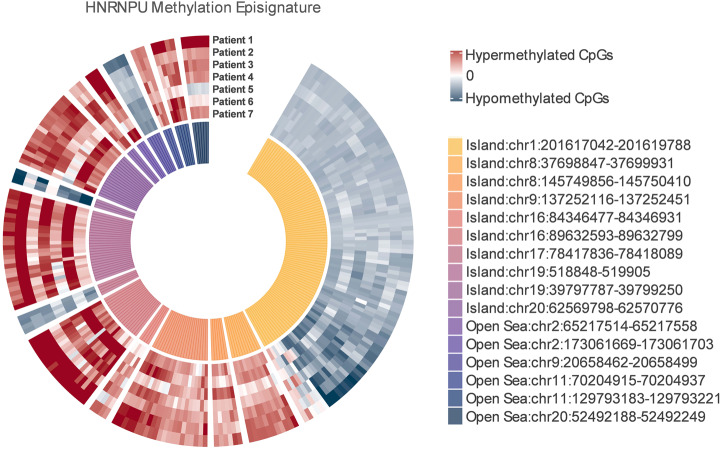
Genomic location and methylation pattern for significantly altered CpGs in *HNRNPU* group. A total of 16 differentially methylated blocks (DMBs) including 10 CpG islands and 6 Open Sea regions were identified as significant among 7 *HNRNPU* patients. Based on methylation values, 12 DMBs were found to be hypermethylated (Island 2, 3, 4, 5, 6, 8, 10 and Open Sea 1, 2, 4, 5, 6) and 4 DMBs (Island 1, 7, 9 and Open Sea 3) were hypomethylated.

Out of 227 DMPs, 16 DMBs including 10 CpG islands and 6 Open Sea regions were identified as significant (Fig. 2). Based on normalised beta-values (normalised value by mean control value [(β-value sample − β-valuecontrol_mean)/σ]), 12 DMBs were found to be hypermethylated and 4 DMBs were hypomethylated. Genes associated with hypomethylated CpGs are *NAV1*, *LRFN1*, *FOCAD* and those related to hypermethylated DMBs are *ADGRA2, LRRC14, LRRC24, RXRA, WFDC1, TPGS1, LINC02245, SLC1A4, PPFIA1, PRDM10*. Two of these genes have been linked with human disease previously, biallelic germline pathogenic variants in *SLC1A4* were reported to cause an autosomal recessively inherited disorder characterised by spastic tetraplegia, thin corpus callosum and progressive microcephaly (MIM 616657) [28–30] and compound heterozygous or homozygous mutations in *FOCAD* were associated with severe congenital liver disease (MIM 619991) [31].

Inspection of hypermethylation/hypomethylation profiles in individual cases (see Fig. 1) showed some variability in the extent of methylation alterations, but there was no obvious relationship apparent between this variability and the type of variant or, for truncating variants, the position of the predicted effect on the *HNRNPU* gene product.

## Discussion

We found evidence of methylation alterations in blood DNA from patients with *HNRNPU*-associated NDD and this, to our knowledge, is the first report of a methylation episignature for *HNRNPU* inactivation by a NGS-based assay. Recently, Rooney et al. [32] published DNA methylation signatures for 9 pathogenic/likely pathogenic and 1 VUS *HNRNPU* variants using data from Illumina EPIC methylation array assay which interrogates fewer CpGs.

The role of members of the heterogeneous nuclear ribonucleoproteins in NDDs (*HNRNPH1, HNRNPH2, HNRNPK, HNRNPR* and *HNRNPU*) and cancer (*HNRNPA1, HNRNPA2B1, HNRNPC, HNRNPD, HNRNPF, HNRNPK, HNRNPR*, and *HNRNPU*) has been the subject of recent investigations but the relationships between the individual function of disease-associated *HNRNPs* and the mechanisms of relevant disorders is not well defined [5, 33]. Our finding of a disordered epigenetic state in peripheral blood DNA from patients with pathogenic variants in *HNRNPU* is consistent with the report of Choufani et al. [16] who described a methylation episignature for *HNRNPK*-associated NDD in 9 individuals. Whereas Choufani et al. [16] used a methylation bead array platform targeting ~850,000 CpGs for methylation profiling, we used a NGS-based assay targeting >2 M CpGs. The difference in methodology and analytical approaches used by Choufani et al. [16] and us limits the detailed comparison of the methylation episignatures from the two conditions. Whereas Choufani et al. [16] identified 429 statistically significant CpG DMPs in their AKS discovery cohort (*n* = 6) using a false discovery rate adjusted *p*-value of 0.05 and a minimum methylation difference of 10%, we employed a *p*-value of less than 0.01 and a more stringent minimum methylation difference of 20% and identified 227 DMPs in our *HNRNPU*-NDD cohort (*n* = 7). However, no overlapping DMBs were found between these 227 DMPs and the 429 CpGs identified from the EPIC array in the *HNRNPK*-NDD cohort (though in the *HNRNPK*-associated episignature of the 429 CpGs identified using the EPIC array, only 178 of these CpGs were present within the target regions of the EPIC-NGS analysis). We note that both in our cohort and in the findings from the *HNRNPK*-AKS cohort studied by Choufani et al. [16], significantly altered DMP events comprised both hypermethylation and hypomethylation alterations (see Fig. 1). However, whereas in our *HNRNPU* cohort, DMPs showed predominantly hypermethylated DMPs (12/16 DMBs), the episignatures from *HNRNPK*-AKS from Choufani et. al. indicated more hypomethylated DMPs than hypermethylated DMPs [16].

The differences in methylation profiling platforms between our investigations and those of Rooney et al. [32] limit a direct detailed comparison of the respective results but there are similarities in the overall episignature patterns with both hyper and hypomethylation alterations. For example, whilst we identified 16 differentially methylated blocks (DMBs) (including 10 CpG islands) among 7 *HNRNPU* patients (12 of which were hypermethylated and 4 were hypomethylated), Rooney et al. [32] identified 18 differentially methylated regions (including 12 CpG islands) with 12 being hypermethylated and 4 hypomethylated. We have previously used EPIC-NGS methodology to identify methylation episignatures in a range of chromatin disorders (e.g., Kabuki syndrome Type 1, *KMT2B*-DYS28, Luscan-Lumish syndrome (*SETD2*) and Rabin–Pappas syndrome (*SETD2*) from healthy controls [17, 26], however a much wider range of NDDs have been studied by methylation array profiling and Rooney et al. [32] compared DNAm patterns in their *HNRNPU* cohort to 56 other NDDs and identified most overlap between the differentially methylated positions within the episignatures for *HNPNPU* with those for velocardiofacial syndrome and BAFopathy cohorts.

The analysis of methylation episignatures in chromatin disorders can often inform likely pathogenicity of variants of uncertain significance (VUSs). Candidate pathogenic *HNRNPU* missense variants are rare [27] and in our cohort all of the patients had a pathogenic loss of function *HNRNPU* variants, so we were not able to formally confirm the utility of DNAm testing for variant interpretation [1]. Nevertheless, the extent of the significant DMPs for our *HNRNPU* cohort suggest that episignature profiling will have clinical utility as a predictor for clarifying pathogenicity of *HNRNPU* VUSs (as described previously for *HNRNPK* variants [16]). In cases of a suspected chromatin disorder in which a VUS is predicted to be non-pathogenic episignature analysis may suggest another diagnosis or suggest the presence of an undetected pathogenic variant [1]. Thus the differential diagnosis of *HNRNPK*-NDD includes Kabuki syndrome and comparison of the methylation signatures for these two disorders would enable them to be distinguished by methylome analysis [16]. Indeed we note that in their recent paper Rooney et al. [32] reported (a) a *HNRNPU* in frame deletion (p.(Glu279del)) which did not demonstrate the *HNRNPU* episignature and (b) an undiagnosed patient who did demonstrate a *HNRNPU* DNAm profile suggestive of *HNRNPU* NDD and was then subsequently found to harbour a candidate pathogenic variant in *HNRNPU* (c.1720_1722delAAG p.(Lys574del)).

The main differential diagnosis of *HNRNPU*-related NDD can be wide due to a number of causes associated with developmental impairment- epileptic encephalopathy (DEE). However, the common differentials include Rett and Angelman syndromes [12] Although Rett syndrome is caused by mutations in the methyl-CpG-binding protein 2 (*MECP2*) it is not associated with a DNA methylation signature and though methylation changes occur in a subset of Angelman syndrome patients, these are generally limited to the imprinted SNURF:TSS-DMR at chromosome 15q11q13 [33, 34]. Therefore, the presence of the relevant DNAm episignature in a child with a clinical suspicion of *HNRNPU*-NDD would be consistent with this diagnosis rather than any other conditions causing DEE including Rett or Angelman syndrome.

*HNRNPU* is abundantly expressed in the developing mouse brain and biallelic loss of *HNRNPU* function was associated with cortical cell death in a genetically-engineered mouse model [35]. Prominent features of *HNRNPU*-NDD include developmental delay, epileptiform seizures, speech and language impairment and behavioural alterations (e.g., autistic features or aggressiveness). Abnormal brain imaging is common (but the range of anomalies is variable) and cardiac and renal structural defects also occur. Transcriptomic studies in the brains of homozygous and heterozygous *HNRNPU*-deficient mouse models demonstrated widespread effects on gene expression, particularly in the homozygote mice affecting multiple signalling pathways including synaptogenesis, neuroinflammation and (cell cycle control [36]. Evidence for disordered RNA splicing (a known role of *HNRNPU*) was detected in *HNRNPU* mutant mice brain cortex [31]. Though RNA splicing is critical for brain development, our findings suggest that the pathogenesis of *HNRNPU*-NDD might also be related to disordered epigenetic regulation of gene expression. Epigenomic and transcriptomic analysis of *HNRNPU* mutant mice might provide further insights into potential disease mechanisms.

Finally, it has been noted previously that rare, apparently healthy, individuals with *HNRNPU* truncating variants may be found in the gnomAD data set (https://gnomad.broadinstitute.org) [12]. This might reflect a lack of detailed phenotypic information or variability of phenotypic expression. However, methylation episignature analysis of such individuals might provide novel insights into genotype-epigenotype-phenotype relationships.

## Supplementary information

Supplementary Table 1

## Data Availability

Data available on request from the authors (subject to patient consent).
