# Supplementary material for: Germline pathogenic variants in HNRNPU are associated with alterations in blood methylome
Source: Eur J Hum Genet. 2023 Jul 5;31(9):1040–7. doi: 10.1038/s41431-023-01422-9 (PMC10474128; doi:10.1038/s41431-023-01422-9)
Supplement: Supplementary file 1 — Supplementary Table 1 [file 41431_2023_1422_MOESM1_ESM.pdf]

[Supplementary Table 1] Sequencing reads and coverage

Sample	Total reads processed	Number of mapped reads (bismarkcov)
**Patient 1**	33,864,153	9,476,634
**Patient 2**	54,583,274	11,312,506
**Patient 3**	19,416,856	8,915,639
**Patient 4**	46,392,754	15,680,389
**Patient 5**	48,211,581	9,990,357
**Patient 6**	34,989,055	9,476,001
**Patient 7**	51,053,405	11,073,213
